# Supplementary material for: Nucleotide signaling pathway convergence in a cAMP‐sensing bacterial c‐di‐GMP phosphodiesterase
Source: EMBO J. 2019 Jul 29;38(17):e100772. doi: 10.15252/embj.2018100772 (PMC6717892; doi:10.15252/embj.2018100772)
Supplement: Supplementary file 1 — Expanded View Figures PDF [file EMBJ-38-e100772-s001.pdf]

Expanded View Figures

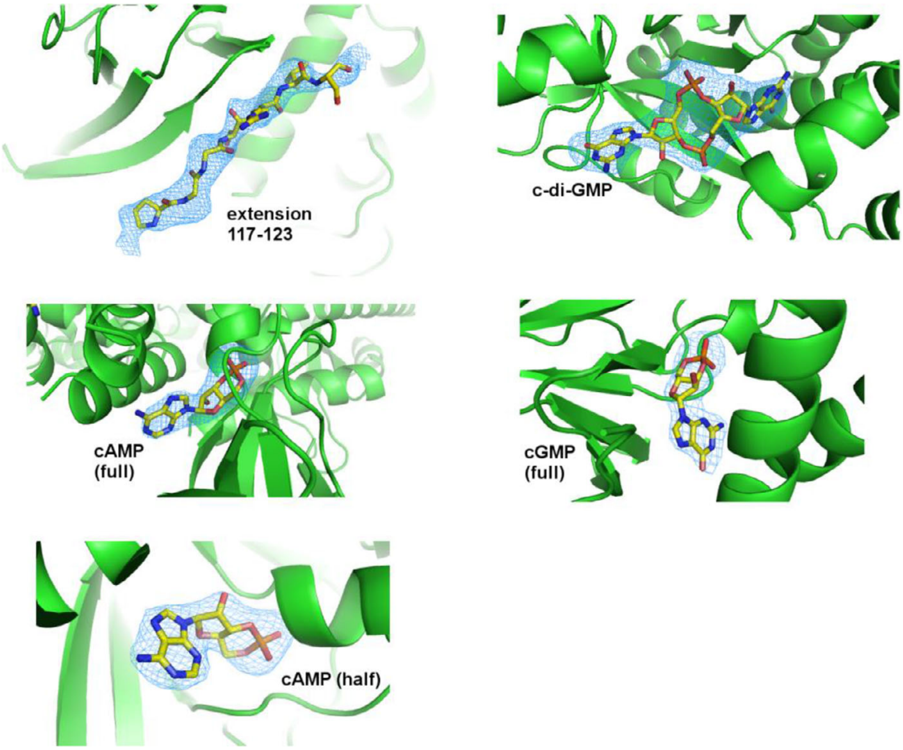

Figure EV1. Omit density features for linker and various ligands.

Maps were calculated (Refmac, FFT) from the respective co-ordinate files with these features removed, and fo-fc density displayed at 3σ. Maps for cAMP were calculated for both the full-length structure and half-occupied sensor form (labeled full and half, respectively).

 © 2019 The Authors

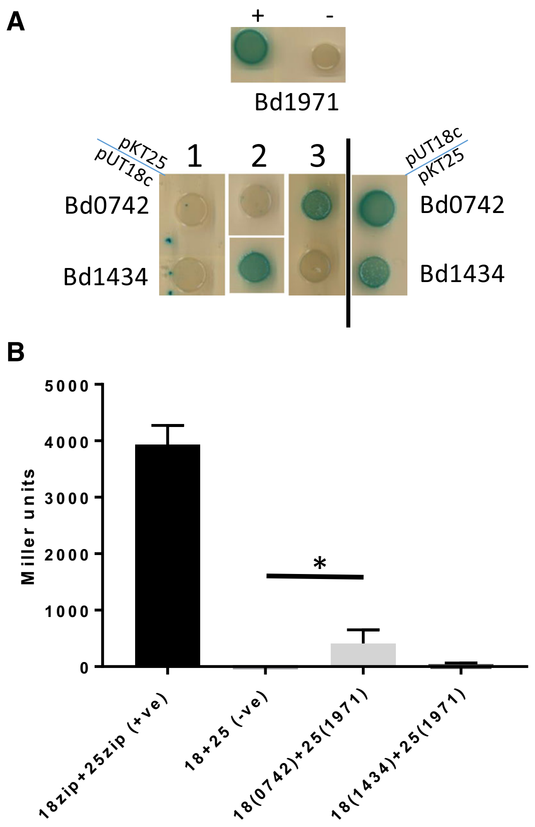

Figure EV2. Bacterial two hybrid (BTH) assays showing weak interactions between Bd1971 and c-di-GMP synthesizing proteins.

A Spot tests of co-transformants of either Bd0742 (DgcB) or Bd1434 (DgcC) with Bd1971. Positive control (+) was co-transformants with pUT18c-zip and pKT25-zip, negative control (—) was the empty vectors. The co-transformants were plated on LB-X-Gal medium with positive interaction resulting in blue coloration. Interactions with Bd1971 in pUT18c were always positive (examples on right of diagram), however with Bd1971 in pUT25, the results were inconsistent (three examples shown 1, 2, 3 on left of diagram), indicative of a weak or transient interaction. Images are representative of at least 3 independent experiments.

B Confirmation of BTH interactions by β-Galactosidase activity assays. The interaction between Bd1971 in pUT25 was significant with diguanylate cyclase Bd0742, but not with Bd1434. *$P > 0.05$ by Student's t-test. Data are from three independent experiments.

Source data are available online for this figure.

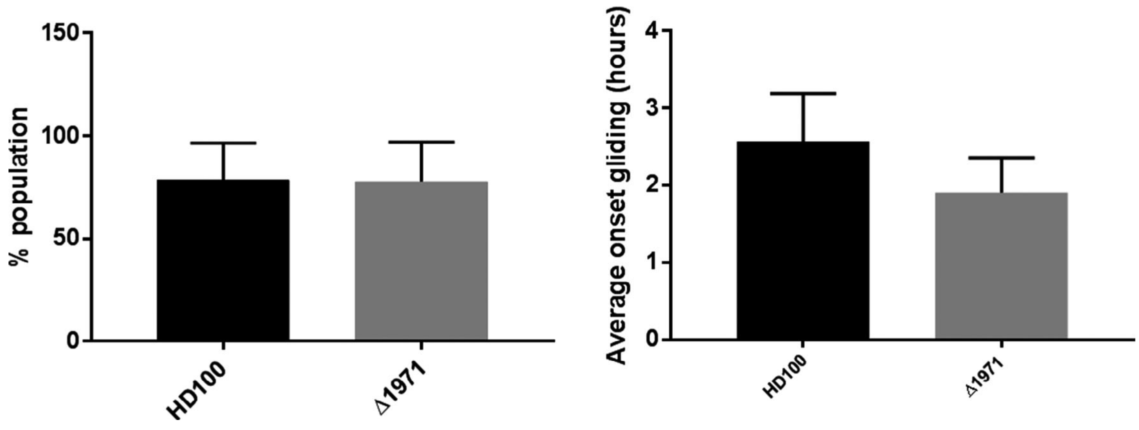

Figure EV3. Percentage of the population of cells demonstrating gliding motility.

Cells were immobilized on 1% agarose Ca/HEPES pads and imaged by phase contrast time-lapse microscopy on a Nikon Ti-E microscope. Time-lapse movies were analyzed, and each cell in a field of view (FOV) was manually scored for motility (in the 6 h period of the movie) and the time from the start of the movie to first observed movement. Data are from two FOV for each of three independent experiments. $n \geq 100$ for each FOV. Error bars are 1 standard deviation.

Source data are available online for this figure.

© 2019 The Authors

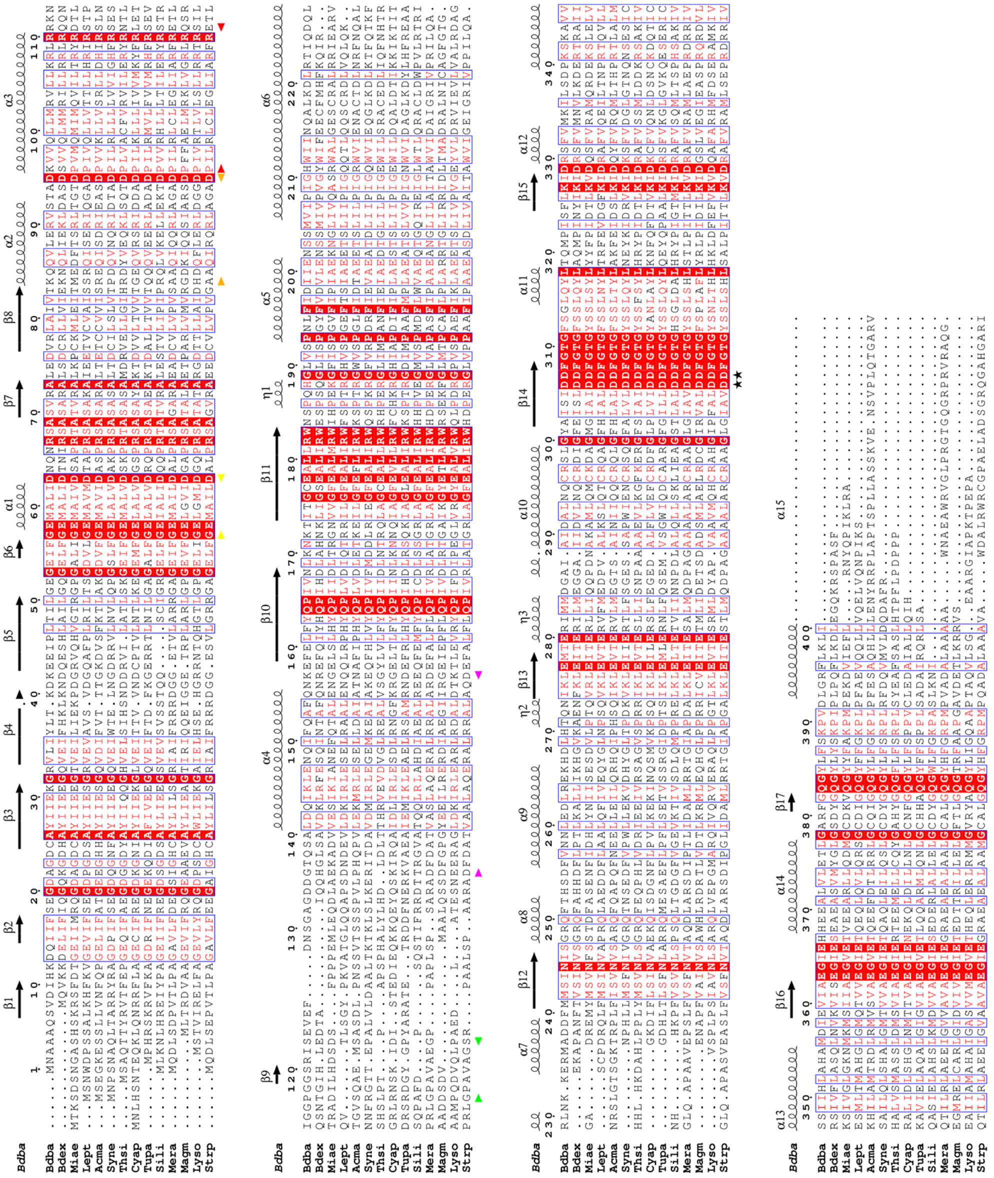

Figure EV4. Annotated sequence alignment of Bd1971 and diverse homologues.

Sequences are abbreviated as follows: Bdba, *Bdellovibrio bacteriovorus* HD100, UNIPROT ID Q6MLN6; Bdex, *Bdellovibrio exovorus* JSS, M4VQ97; Miae, *Micavibrio aeruginosavorus*, G2KM87; Lept, *Leptolyngbya* sp. PCC 7375, K9F361; Acma, *Acaryochloris marina*, B0CEC6; Syne, *Synechocystis* sp. strain PCC 6803, Q55427; Thsi, *Thiorhodospira sibirica*, G4E2D0; Cyap, *Cyanobacterium aponinum*, K9Z4H2; Tupa, *Turneriella parva*, I4B4Z1; Sili, *Sideroxydans lithotrophicus*, D5CLE0; Mera, *Methylobacterium radiotolerans*, B1M546; Magm, *Magnetospirillum magneticum*, Q2W2M8; Lyso, *Lysobacter dokdonensis*, A0A0A2WD48; Strp, *Streptomyces purpurogeneiscleroticus*, A0A0N0B6Y0. The alignment was prepared using ESPRIPT (secondary structure calculated using the cGMP complex co-ordinates) and regions delineated by start/end arrows correspond to those used in Fig 3A (P-loop, 57–63, yellow; hinge, 84–95, orange; C-helix, 96–111, red; extension, 117–123, green; linker, 136–157, magenta). The two active site residues, D306 and D307, are labeled with a star character.

© 2019 The Authors
